# Self-healing of type B acute aortic dissection after aortic valve replacement: a case report

**DOI:** 10.1186/s13256-023-04030-9

**Published:** 2023-07-05

**Authors:** Ling Jiang, Xiangrong Xie, Jun Wei

**Affiliations:** 1 Department of Cardiology, The First Affiliated Hospital of Wannan Medical College (Yijishan Hospital of Wannan Medical College), 2# West Zhe Shan Road, Wuhu, 241001 Anhui People’s Republic of China; 2 Department of Cardiac Surgery, The First Affiliated Hospital of Wannan Medical College (Yijishan Hospital of Wannan Medical College), 2# West Zhe Shan Road, Wuhu, 241001 Anhui People’s Republic of China

**Keywords:** Aortic dissection, Aortic intramural haematoma, Aortic valve regurgitation, Aortic valve replacement, Case report

## Abstract

**Background:**

As a life-threatening and serious condition, aortic dissection (AD) is divided into type A and B according to its association with the ascending or descending aorta. Type A AD is often accompanied by aortic regurgitation, while type B dissections are rarely accompanied by severe aortic regurgitation.

**Case presentation:**

We present a 71 year-old Chinese man with a rare case of type B AD with severe aortic insufficiency, who self-healed after 1 year of an aortic valve replacement. He complained of chest tightness and abdominal pain. Due to poor cardiac function, he underwent aortic valve replacement before intervening on the dissection. The operation was successful, and the dissection was treated conservatively. During the 1-year follow-up, his chest tightness improved, and the type B dissection was healed. His general condition is considerably improved.

**Conclusions:**

In type B AD combined with severe aortic insufficiency, aortic valve replacement should be prioritized. This is potentially explained by the aortic root activity and pulse pressure difference.

## Background

Aortic dissection (AD) is defined as disruption of the medial layer provoked by intramural bleeding, resulting in separation of the aortic wall layers and subsequent formation of a true lumen (TL) and an false lumen (FL) with or without communication [1]. Existing studies^−^ have demonstrated that the incidence of AD is about 6 in 100,000 per year [2]. Its incidence is higher in men than in women and common risk factors for AD include the aortic aneurysm, long-term arterial hypertension, and age [3]. It can be classified into Stanford type A AD or B AD depending on whether the ascending or descending aorta are involved. Acute type A dissection often needs a swift open surgical repair; otherwise the patient may die from complications related to the dissection, including rupture of the aorta, aortic regurgitation, insufficient organ perfusion, or acute heart failure [4]. As the understanding of the mechanism of type B AD improves and the advancement of endovascular treatment for distal dissection, the boundary between complicated and uncomplicated type B dissection has gradually become blurred, and the optimal treatment method for type B dissection is still actively being explored [5]. There are few reports of patients with a type B dissection complicated with aortic valve insufficiency. Here, we describe a case of type B AD with severe aortic insufficiency, in which the dissection healed 1 year after aortic valve replacement. The experience summary, postoperative management, surgical strategy, and medical therapy are reported in the following sections.

## Case presentation

**Fig. 1:**
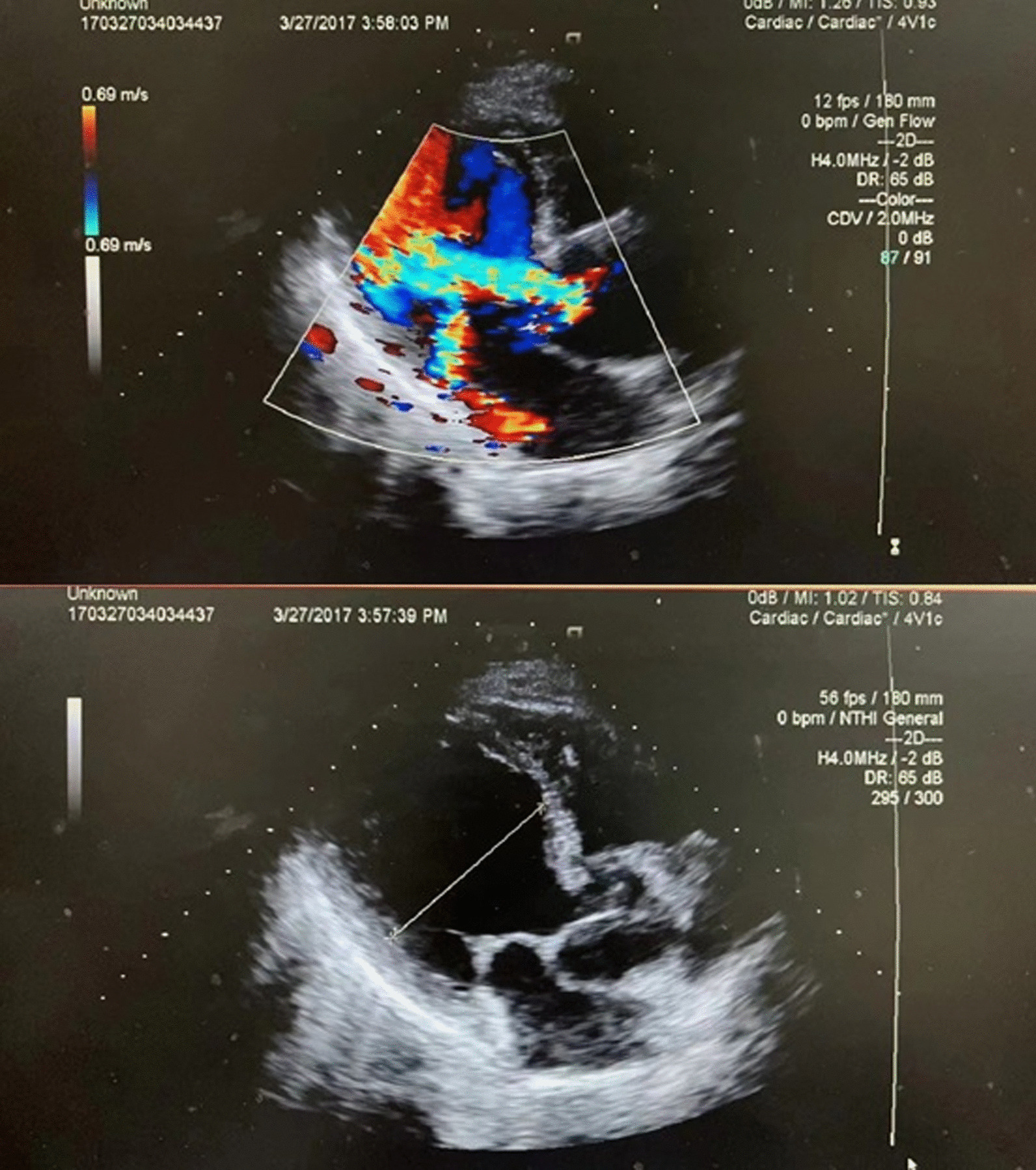
The patient’s echocardiographic results in 2017

**Fig. 2:**
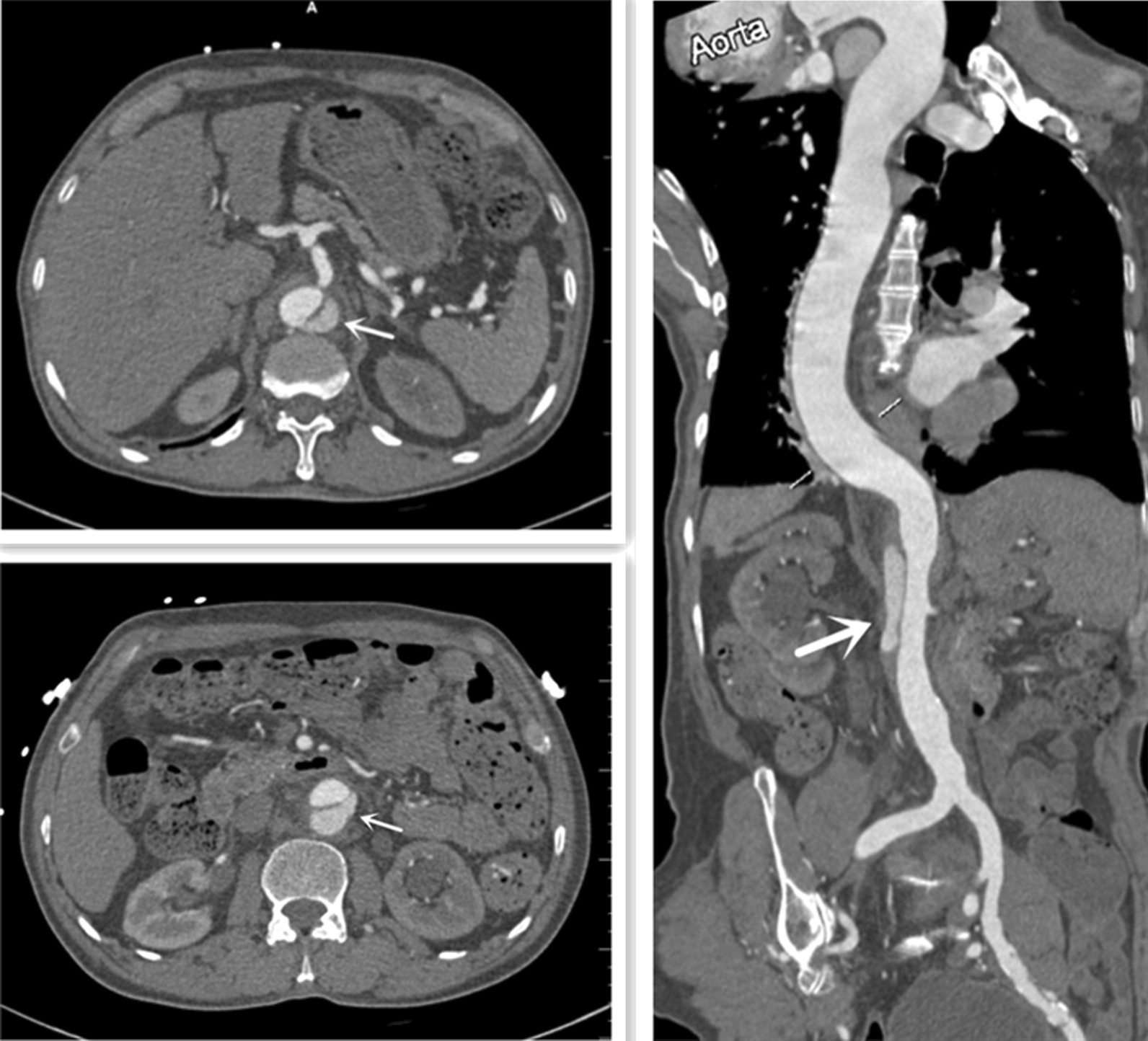
Initial the thoracoabdominal aorta computed tomographic angiography showing penetrating atherosclerotic ulcer in the descending aorta. The white arrow refers to the false lumen at the level of abdominal aorta and Mesentery artery before operation

**Fig. 3:**
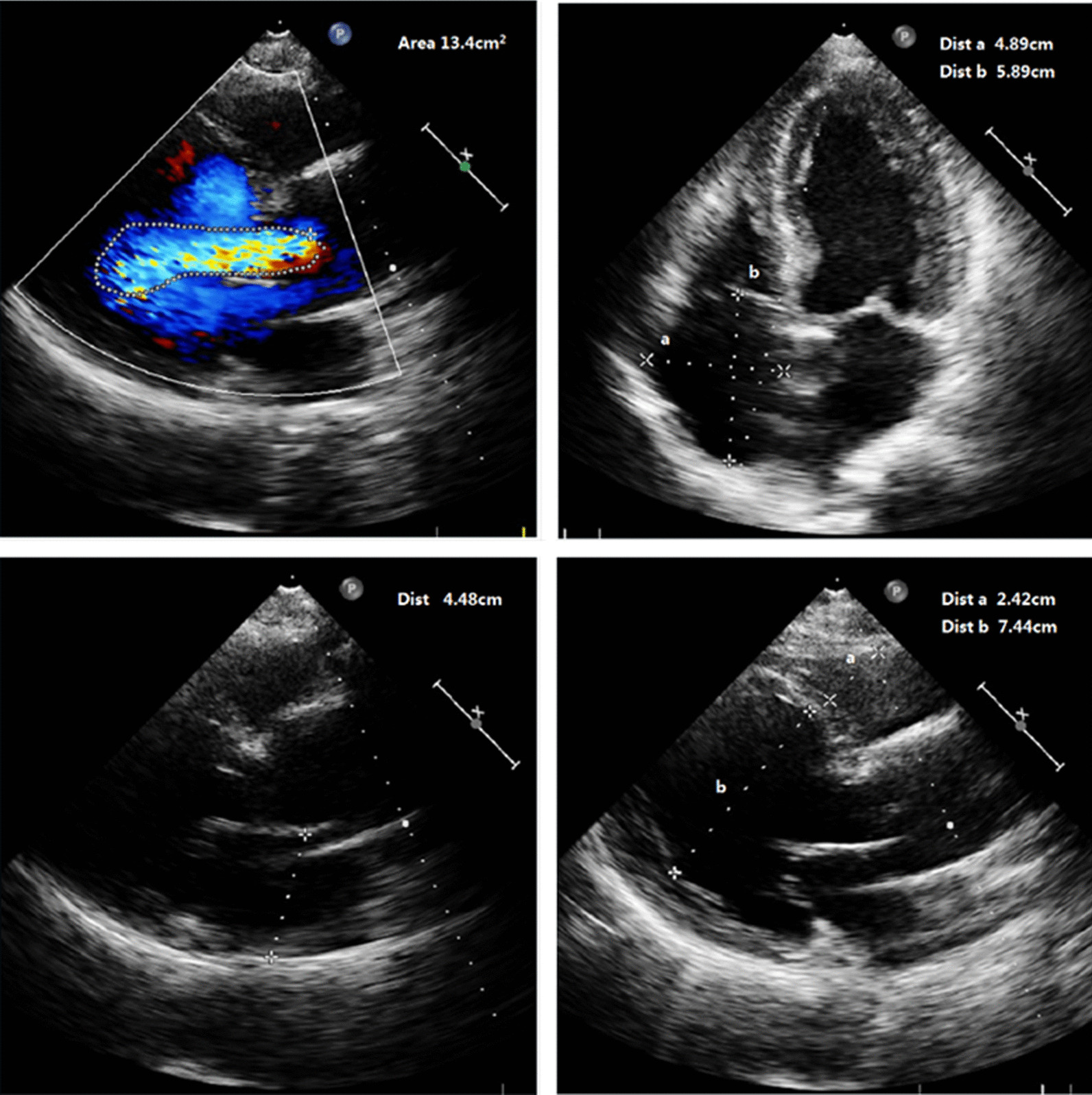
Preoperative echocardiography

A 71 year-old Chinese man with past medical history of hypertension and moderate to severe aortic insufficiency presented to the hospital due to chest tightness & abdominal pain for four days. He was admitted to the geriatrics department of our hospital on December 22, 2020. This patient had a history of hypertension, and took carvedilol and nifedipine controlled-release tablets for long-term antihypertensive treatment. His blood pressure was usually well controlled and he had regular outpatient follow-up. The patient's cardiac color Doppler ultrasound in 2017 revealed moderate to severe aortic regurgitation, with an enlarged inner diameter of the ascending aorta (44 mm). Cardiac enlargement (left atrium 44 mm, right atrium 66×50 mm, left ventricle 70 mm, right ventricle 25 mm), left ventricular ejection fraction 60% (Fig. 1). The patient did not present other diseases, such as type 2 diabetes. Additionally, We also did not find a history of secondary hypertension, connective tissue diseases such as Marfan syndrome, or acute aortic syndrome in patients. Vitals observed at the time of admission: Temperature 37.5 °C, pulse 71 beats per minute at rest, respiratory 18 breaths per minute, BP 151/61 mmHg. Regular heartbeats and a systolic murmur of the aortic valve could be heard. No additional heart sounds and pericardial friction rubs were heard. Sinus rhythm, left ventricular hypertrophy, and ST-T segment changes were indicated by the ECG. Acute aortic syndrome was suspected based upon symptoms and history. A CTA scan with contrast agent revealed aneurysm of the innominate artery arising from the aortic arch and an acute uncomplicated type B AD with penetrating atherosclerotic ulcers, involving the descending aorta. Contrast agent of the descending aorta and the right external iliac artery displayed a limited sac-like shadow, with the largest cross-sectional area located at the level of the abdominal aorta and mesenteric artery (area of 3.0*1.6 cm) (Fig. 2). The brachiocephalic trunk of the aortic arch was dilated, but the celiac trunk, left subclavian artery, left common carotid artery, and bilateral renal artery openings were not involved. The patient was then immediately transferred to the Cadiac Surgery department. Echocardiography revealed an aneurysmal dilatation of the ascending aorta and ascending aortic sinus (inner diameter of about 47 mm), cardiac enlargement (LA 45 mm, RA 59*49 mm, LV 74 mm, RV 24 mm), severe aortic valve insufficiency, and left ventricular ejection fraction of 54% (Fig. 3). The patient was treated with urapidil infusion, carvedilol 10 mg bid, and nifedipine controlled-release tablets 30 mg qd, with a target blood pressure of systolic 100-130 mmHg, mean arterial pressure 60-70 mmHg, and heart rate 60–80 beats/min. His symptoms were markedly improved as conditioned gradually stabilized. Repeat CT Chest angiography showed stable dissection without any further extension.

After consultation with the experts in the field of cardiothoracic surgery, taking into consideration the patient's age and the relative contraindications to long-term anticoagulation therapy in the case of AD, it has been planned to perform an open surgical procedure for aortic valve replacement with a biological valve prosthesis after 20 days. During the surgical operation, a degeneration of the aortic valve was found, but there was no obvious coronary valve prolapse, mild valve margin thickening or aortic valve insufficiency, therefore an aortic valve bioprosthetic replacement was performed after valve leaflet removal. Pathology revealed uneven thickness of fibrous connective tissue with collagenization and myxoid degeneration. The patient was discharged 10 days after surgery.

**Fig. 4:**
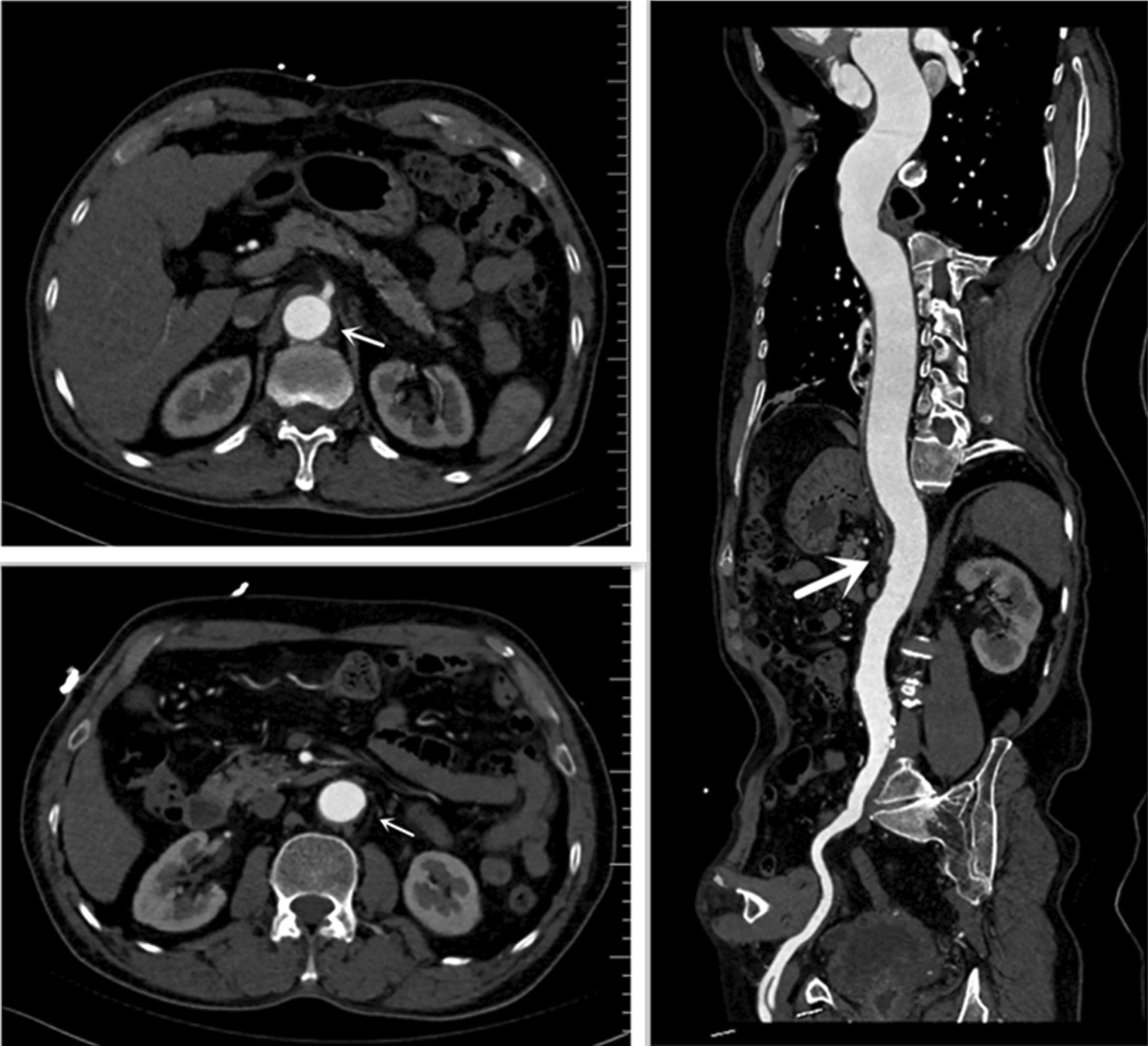
1 year postoperatively, computed tomographic angiography of the thoracoabdominal aorta showed healing of the dissection and the blood vessels were intact. The white arrow indicates that the false lumen at the level of abdominal aorta and Mesentery artery has healed 1 year after operation

**Fig. 5:**
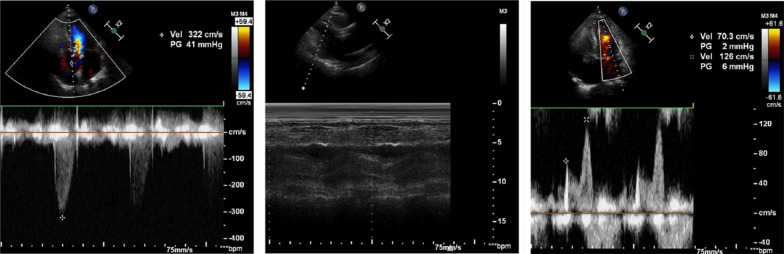
1 year follow-up after operation, echocardiography results

**Fig. 6:**
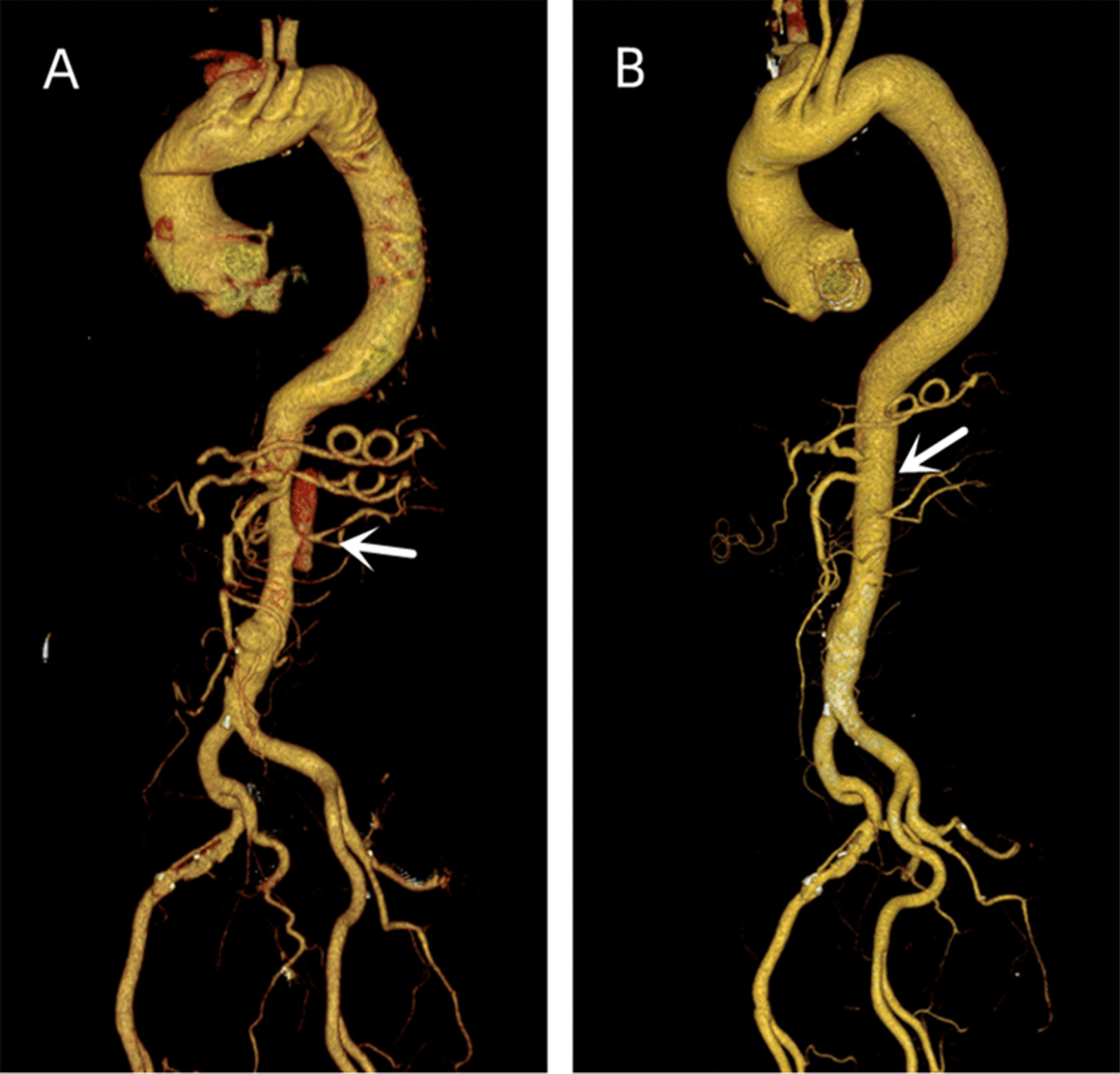
Three dimensional reconstructions at: **A** Initial AD, **B** Postoperative one year re-examination. The white arrow refers to the three-dimensional view of the false lumen at the abdominal aorta before surgery and the abdominal aorta that has healed one year after surgery

On the follow-up CTA scan 1 year after the initial event, his dissecting ulcers had disappeared, the hematoma had largely resorbed and his aorta looked completely normal (Fig. 4). Echocardiography revealed the postoperative status of the aortic valve replacement, atrial enlargement (LA 43 mm, RA 59*39 mm, LV 51 mm, RV 20 mm), and a left ventricular ejection fraction of 66% (Fig. 5). The patient's heart was significantly remodeled. Three dimensional reconstructions revealed a favorable prognosis (Fig. 6). He is doing well, with no new complaints and his blood pressure is well controlled.

## Discussion

Risk factors for type B AD are the same as those for type A and fall into 2 broad categories: conditions associated with decreased aortic wall strength and factors involved in increased stress of the aortic wall [6]. Increased stress of the aortic wall is significantly associated with iatrogenic injury, pregnancy, cocaine or stimulant use, deceleration trauma, hyperlipidemia, smoking, atherosclerosis, and uncontrolled arterial hypertension. It is worth noting that hypertension is the most commonly identified risk factor for type B AD. In parallel, genetic disorders of the connective tissue, such as Marfan syndrome, and inflammatory vasculitis, including giant cell arteritis and Takayasu arteritis contribute to decreased aortic wall strength.

Type B AD can be further classified as complicated or uncomplicated according to the existence of at least one additional symptom, such as malperfusion of the lower extremity vasculature, spinal viscera, or kidneys, uncontrollable hypertension, rapid aortic expansion, refractory pain, or aortic rupture. The presence of at least one of the abovementioned symptoms define acute complicated type B AD [7]. Currently, the mortality for type B AD has been stable at approximately 13%, while acute complicated type B AD may require emergency surgery. The distinction between complicated and uncomplicated type B AD is becoming blurred, and complex surgery is replaced by a thoracic endovascular aortic repair. Indeed, correction of type B AD may promote remodeling of the aorta and reduce late aortic-related death and complication rates [7, 8]. Current guidelines recommend that uncomplicated type B AD should be treated with drugs—the main purpose of which is to reduce the shear stress of the aortic lesion through decreasing myocardial contractility and blood pressure [1].

The case herein reported was an uncomplicated type B AD with severe aortic regurgitation. This is a very rare case in which there are no guidelines recommending treatment strategies. The patient's heart was enlarged and his cardiac function was impaired. We initially used pharmacological treatments to relieve his pain, reduce arterial blood pressure, and improve cardiac function. During this period, the patient underwent aortic valve bioprosthetic valve replacement surgery. The patient routinely took 3 mg qd of warfarin for anticoagulation therapy postoperatively, with a target INR of 2.0–2.5 for a duration of six months. After 1 year, the CTA scan results were basically normal, and his heart underwent reverse remodeling. This outcome may be attributed to a tight control of his blood pressure, which directly reduces aortic wall stress. At the same time, the healing of dissection could also be attributed to the aortic valve replacement, which may directly reduce aortic root motion and indirectly reduce aortic wall shear force. This is corroborated by a previous study that demonstrated that aortic valve replacement due to aortic regurgitation could significantly reduce the postoperative aortic root motion and decrease the incidence of aortic wall dissection induced by mechanical stress [9]. Therefore, we believe that the rapidly increased blood pressure could effectively enhance the velocity of the aorta blood flow, increase the aortic root mobility, elevate the influence of geometric factors in aortic segments, and increase the wall shear stress of the vascular wall at the distal portion of the aortic arch, thus resulting in the tear of the aortic intimal layer. Moreover, we hypothesize that aortic valve replacement may reduce aortic dilatation and afterload to enhance forward blood flow in the TL and heal the FL.

## Conclusion

We can conclude from this case that aortic valve replacement is of vital importance to the prognosis of patients of AD concomitantly with severe aortic valve insufficiency, and blood pressure management is fundamental to treatment. There are no reported cases similar to type B AD accompanied by severe aortic dysfunction currently. A review of this case may allow clinicians to optimize clinical treatment strategies for type B AD.

## Data Availability

All data can be found at the Department of the First Afliated Hospital of Wannan Medical College.

## Abbreviations

AD: Aortic dissection
CTA: Computed tomographic angiography
TL: True lumen
FL: False lumen
LA: Left atrium
RA: Right atrium
LV: Left ventricle
RV: Right ventricle
